# Association between mass media exposure and infant and young child feeding practices in India: a cross-sectional study

**DOI:** 10.1038/s41598-023-46734-4

**Published:** 2023-11-07

**Authors:** Dhriti Dhawan, Ramya Pinnamaneni, K. Viswanath

**Affiliations:** 1 Dana-Farber Cancer Institute, 450 Brookline Avenue, LW 601, Boston, MA 02215-5450 USA; 2 Harvard T.H. Chan School of Public Health, Boston, MA USA

**Keywords:** Disease prevention, Health policy, Nutrition, Public health, Quality of life

## Abstract

The first two years of life is a critical window for good nutrition. Promoting infant and young child feeding (IYCF) practices in the first two years can help improve child survival and promote healthy growth and development. Assessment of IYCF practices is important, especially in developing countries like India where optimal IYCF practices can potentially prevent 12% of all deaths under 5 years of age, to promote awareness and intervene appropriately. The objective of our study is to generate evidence for the association between different types of mass media and appropriate IYCF practices in India, including optimal breastfeeding and appropriate complementary feeding practices. A positive association between them can point to intervention at scale. We analyzed data from India’s National Family Health Survey 5 (NFHS-5), 2019–2021. Multivariable logistic regression was used to examine the association of appropriate IYCF practices with mass media exposure. After controlling for demographics and socioeconomic status, the analyses showed that, overall, women who had exposure to television followed by newspaper and movies, had higher odds of adopting the recommended IYCF practices. The results also showed that the association of media exposure varied for different IYCF practices by geography. For instance, in the rural areas, television exposure was positively associated with all the IYCF practices, but in the urban areas, television exposure was positively associated with only early initiation of breastfeeding [OR 1.25; (95% CI 1.1–1.42)]. The study strengthens our understanding that an appropriate selection of mass media channels for intervention programs can promote IYCF practices at scale. Appropriately selecting the type of mass media to create awareness about different IYCF practices, in specific urban–rural settings, could help customize intervention programs to successfully influence IYCF behaviors.

## Introduction

Right food at the right time helps children grow and develop to their full potential. The first 1000 days of life, starting from the mother’s pregnancy to the child’s second birthday, is a critical window for good nutrition. Undernutrition in the first 2 years of age increases the risk of mortality, developing chronic diseases later in life, impaired cognitive development as well as diminished work capacity^1,2^. Globally, 45% of deaths among children under 5 years of age are linked to undernutrition, of which two thirds are attributed to sub-optimal infant and young child feeding (IYCF) practices, especially during the first year of life^3–5^. In developing countries like India, optimal IYCF practices can potentially prevent 12% of all deaths under 5 years of age^4^.

The World Health Organization (WHO) and the United Nations Children's Fund (UNICEF) recommendation for optimal IYCF includes breastfeeding practices like early initiation of breastfeeding within the first hour after birth (EIBF), exclusive breastfeeding (EBF) from birth to six months of age, and complementary feeding practices like timely introduction of nutritionally adequate and safe complementary foods at six months while continuing breastfeeding up to two years of age or beyond^5^. Promoting optimal IYCF practices can help improve child survival as well as promote healthy growth and development.

Prelacteal feeding, a common practice in India, includes giving something other than breastmilk to the newborn, such as honey or gripe water, and breastmilk substitutes like “baby formula”, during the first three days of life^6,7^. Feeding newborns anything other than breastmilk is highly discouraged under the global and national IYCF guidelines^7–9^. Prelacteal feeding also means that the infant is not exclusively breastfed. Moreover, prelacteal feeding can cause a delay in breastfeeding initiation as well as colostrum deprivation^6,10^. EIBF is important for both the mother and the child because the first breastmilk contains colostrum, which is highly nutritious and has antibodies that protect the newborn from diseases. EIBF also encourages bonding between the mother and her newborn, facilitating the production of regular breastmilk^11^.

Breastmilk is an uncontaminated nutritional source, which contains all the nutrients needed by children in the first six months of life. Hence, it is recommended that, for the first six months of their life, children are given nothing but breastmilk and be exclusively breastfed^12,13^. EBF is the foundation of optimum infant nutrition and has been found to reduce the risk of diarrheal diseases, upper respiratory tract infections and obesity in later life of the child^14^. Many studies have documented beneficial effects of breastfeeding on infant’s nutritional status and mortality^15^. Epidemiological evidence suggests that practicing both EIBF and EBF practices in combination is associated with higher reduction in mortality compared to practicing them individually^16,17^.

After the first six months, appropriate complementary feeding should be started for the child because breastmilk is no longer sufficient to meet the nutritional needs of an infant^5^. Timely introducing complementary foods like safe and nutritionally balanced solid, semi-solid or soft foods in addition to breast milk for children between 6 and 23 months, is essential for child growth and provides the foundation for good health throughout life^18^. Moreover, infants and young children need to be fed with minimum acceptable diet (MAD) to ensure appropriate growth and development^19^. According to the WHO, MAD is a combination of dietary diversity and minimum meal frequency and is different for breastfed and non-breastfed children^20^. We have limited our study to explore the MAD of breastfed children, which includes a variety of foods like fruits and vegetables including those rich in vitamin A, to ensure that nutrient requirements are met.

Despite the numerous benefits and advantages of appropriate IYCF practices, the prevalence of EBF, EIBF and appropriate complementary feeding in India remain low^18,21^. Poor IYCF practices lead to high prevalence of undernutrition like wasting and stunting^19^. The WHO/UNICEF emphasize the need for policies, including improvement of communication strategies, to increase awareness and uptake of, and support for IYCF practices. They also suggest that communication strategies should be appropriately selected for the target population and take their literacy levels and media access into consideration^12^.

### Mass media

Mass media are critical in promoting health because their widespread penetration promotes broad reach to key audiences across geographic and other social boundaries, and the exposure to specific messages in the media is known to shape public knowledge, attitudes, beliefs, and behaviors^22^. Over the past few decades, media campaigns have been used to influence behavioral changes^23,24^. Creating awareness through mass media can encourage positive and discourage negative health related behaviors across populations^25–28^.

Literature suggests that altering the information and message environment, maximizing exposure to campaign messages, creativity of the messages and influencing the determinants of the behavior targeted in the campaign can ensure the success of mass media public health campaigns^24^. These factors can be extremely valuable in designing effective public health campaigns and achieving the desired goals.

To improve nutritional outcomes for children, pregnant women, and lactating mothers in India, in March 2018, the Ministry of Women and Child Development (MoWCD), Government of India launched a flagship program called the Prime Minister’s Overarching Scheme for Holistic Nourishment (POSHAN) Abhiyaan or the National Nutrition Mission^29^. One of the objectives of the mission is to build knowledge, attitudes, and behavioral intent about overall nutrition amongst their target populations. To achieve this, the mission is utilizing various platforms, including mass media, to create a people’s movement or *Jan Andolan.* The objective of our study is to generate evidence for the association between different types of mass media and appropriate IYCF practices in India, including optimal breastfeeding and appropriate complementary feeding practices, which are also the key themes of POSHAN Abhiyaan. Existing survey datasets do not capture data related to exposure specifically to IYCF messaging through mass media. Nevertheless, the inclusion of national campaigns on mass media by the MoWCD in both 2006 and 2018 can be seen as indicative of potential exposure to IYCF messaging^29–31^. Our findings can inform public health campaigns like POSHAN Abhiyaan to adjust their communication strategies to successfully achieve their goals to increase the adoption of appropriate IYCF behaviors.

## Methods

### Data source

This study uses the women’s dataset from India’s National Family Health Survey (NFHS) 5, 2019–2021. The NFHS is a series of nationally representative cross-sectional surveys that provide data on a range of demographic, socioeconomic, maternal and child health outcomes; reproductive health; and family planning. NFHS-5 was conducted in two phases and gathered information from 636,699 households, 724,115 women aged 15–49 years and 101,839 men aged 15–54 years, with a response rate of 98%, 97% and 92%, respectively. Only the women who gave birth in the last 36 months were included in our analyses. The final analytical sample included 122,458 women.

### Sample design

A sample design representative of the national, state/union territory and district level was adopted in each round of the survey. Every district was stratified into urban and rural areas. The rural stratums were sub-stratified based on the village population and the percentage of population belonging to the scheduled castes (SC) and scheduled tribes (ST). A sample of villages was selected as Primary Sampling Units (PSUs) within each rural sampling stratum. Prior to the PSU selection, the PSUs were sorted according to the literacy rate of women (only girls and women who were 6 years or older were included in the literacy rate calculation). Similarly, a sample of Census Enumeration Blocks (CEBs) was selected as PSUs within each urban sampling stratum. Prior to the selection of the PSUs, they were sorted according to the percentage of SC/ST population. In the second stage, twenty-two households were selected from each cluster with an equal probability systematic selection from a newly created list of households in the selected PSUs.

### Outcome variables

The outcome variables in this study included the optimal IYCF practices for the women’s most recent pregnancy in the last 36 months, comprising of no prelacteal feeding, EIBF and EBF, and appropriate complementary feeding practices comprising of timely introduction of complementary foods and Minimum adequate diet (MAD) for breastfed children ages 6–23 months.

### Optimal breastfeeding practices

*No Prelacteal feeding* Prelacteal feeding status was measured for children below 36 months of age. If a mother responded that her child was not given anything to drink other than breastmilk in the three days after the delivery, the child was categorized as “received no prelacteal feeding”.

*Early initiation of breastfeeding (EIBF)* EIBF was measured for children below 36 months of age. The child was categorized as to have “initiated early breastfeeding” if the child was put to the breast immediately after birth.

*Exclusive breastfeeding (EBF)* EBF was measured for currently breastfeeding children below six months of age. Children were categorized as “exclusively breastfed” if their mothers reported that they consumed nothing but breastmilk the previous day.

### Appropriate complementary feeding practices

*Timely introduction of complementary foods* Timely introduction of complementary foods was measured for mothers with children between six to eight months of age. If the mother said that her child had consumed some solid, semi-solid, or soft foods the previous day, in addition to breastmilk, the child was categorized as having timely introduced complementary foods.

*Minimum acceptable diet (MAD) for breastfed children ages 6–23 months* MAD was measured for breastfeeding children between 6 and 23 months of age, who had received four or more food groups (i.e., solid, or semi-solid foods from at least four food groups not including the milk or milk products food group), and a minimum meal frequency, that is receiving solid or semi-solid food at least twice a day for breastfed infants 6–8 months and at least three times a day for breastfed children 9–23 months, the previous day.

Timely introduction of complementary foods and MAD for breastfed children ages 6–23 months variables were used as a proxy for appropriate complementary feeding practices.

### Independent variables

Level of exposure to different types of media sources, like newspaper or magazine, radio, television, and movies at a cinema hall was measured by asking the women to indicate how often they read a newspaper or a magazine, listened to a radio, watched television, and went to a cinema hall or a theatre to see a movie. Women who responded, “at least once a week” (or “at least once a month” for movies) were considered to have “high exposure” to that media. Women who responded, “less than once a week” (“less than once a month” for movies) or “not at all” were considered to have “some exposure” and “no exposure”, respectively. Cumulative exposure to mass media was also measured by combining all four media types. Women who had “no exposure” to any type of media were categorized as having “no exposure” to any media. If they had “some exposure” or “high exposure” to one or more types of media, they were categorized as having “some/high exposure” to any media.

### Covariates

Standard and commonly used questions were used to measure demographic and social factors: age of the mother, education, wealth status, caste, and religion.

### Statistical analysis

Frequencies of key variables were obtained to describe the study sample. In this study, we conducted multivariable logistic regression to examine the associations between exposure to different types of media: newspaper or magazine, radio, television, and movies, and the recommended IYCF practices: no prelacteal feeding, EIBF, EBF, timely introduction of complementary foods and MAD for breastfed children ages 6–23 months. All the regression models were controlled for other media variables and the covariates. We also examined the association between cumulative mass media exposure (hereafter “any media”) and the recommended IYCF practices, controlling for the covariates. The data were analyzed and weighted using R version 4.3.1 (2023.06.16). Survey weights were included in the analysis and the survey-weighted logistic regression was performed using the R survey package. All the analyses were conducted in 2023.

## Results

### Descriptive characteristics

Our analysis of the NFHS-5 dataset showed that, in India, 84.6% mothers reported that their child *did not* receive any prelacteal feed (N = 116,908). However, only 41.7% mothers reported that their child was put to the breast immediately after birth (N = 122,458), hence initiating early breastfeeding. At the time of the survey, amongst the mothers who were currently breastfeeding their infants under six months of age, only 63.8% reported that their infant was being exclusively breastfed (N = 22,839). The rate of EBF was higher in the rural areas compared to the urban areas. Amongst the mothers who were currently breastfeeding their six to eight months old infants, only 45.9% reported timely introduction of complementary foods (N = 10,809). Rates of timely introduction of complementary foods were 8% higher in the urban areas compared to rural areas. Furthermore, merely 11% mothers reported that their 6–23 months old breastfeeding infants were being fed with a MAD (N = 53,144). (Table 1) This indicates that, apart from not giving prelacteal feed to infants, the prevalence of optimal IYCF practices is low in India, especially in rural India.

**Table 1:** Socioeconomic, demographic, media exposure and optimal IYCF practices characteristics among women who gave birth in the last 36 months, in the National Family Health Survey 5 (2019–2021).

Indicators	Total (%), N = 122,458	Rural total (%), N = 97,409	Urban total (%), N = 25,049
Population characteristics			
Age of mother (years)			
15–19	4.4	5.0	2.5
20–24	35.7	38.1	29.3
25–29	37.6	36.7	39.9
30–34	16.0	14.3	20.7
35–39	5.2	4.8	6.4
40–44	1.0	0.9	1.1
45–49	0.2	0.2	0.1
Educational attainment			
No education	19.0	22.3	10.0
Incomplete primary	11.4	12.5	8.4
Incomplete secondary	49.9	50.3	48.8
Complete secondary	2.0	2.0	1.9
Higher	17.7	12.9	30.9
Wealth			
1st quintile (poorest)	23.5	30.7	4.1
2nd quintile	21.4	26.1	8.6
3rd quintile	19.6	20.6	16.9
4th quintile	19.0	15.2	29.3
5th quintile (richest)	16.5	7.5	41.2
Urbanicity			
Urban	26.8	0.0	100.0
Rural	73.2	100.0	0.0
Caste			
Schedule caste	23.3	24.4	20.2
Schedule tribe	10.1	12.3	4.1
Other backward class	43.7	43.5	44.4
None of them/general	22.9	19.8	31.3
Religion			
Hindu	79.5	81.5	73.8
Muslim	16.2	14.2	21.6
Christian	2.1	2.0	2.3
Sikh	1.3	1.4	1.1
Other	1.0	1.0	1.3
Media			
Newspaper or magazine use			
No exposure	70.3	75.8	55.2
Some exposure	18.7	16.5	24.6
High exposure	11.1	7.7	20.2
Radio use			
No exposure	88.1	89.2	84.8
Some exposure	8.6	8.0	10.2
High exposure	3.3	2.7	4.9
Television use			
No exposure	31.4	37.6	14.7
Some exposure	20.0	20.5	18.7
High exposure	48.5	41.9	66.6
Movie use			
Some exposure	91.2	94.3	83.0
High exposure	8.8	5.7	17.0
Any media use			
No exposure	22.5	29.0	8.8
Some/high exposure	77.5	71.0	91.2
IYCF practices			
No prelacteal feeding^1^ (N = 116,908)	84.6	85.3	82.5
Early initiation of breastfeeding^2^ (N = 122,458)	41.7	40.7	44.7
Exclusive breastfeeding^3^ (N = 22,839)	63.8	65.1	59.8
Timely introduction of Complementary foods^4^ (N = 10,809)	45.9	43.8	52.0
Minimum acceptable diet for breastfed children ages 6–23 months^5^ (N = 53,144)	11.0	10.8	11.8

^1^Children did not receive a prelacteal feed. ^2^Children under age 3 years breastfed within one hour of birth. ^3^Children under age 6 months receiving nothing but breastmilk. ^4^Children age 6–8 months receiving solid or semi-solid food and breastmilk. ^5^Breastfed children receiving 4 or more food groups (solid or semi-solid foods from at least four food groups not including the milk or milk products food group) and a minimum meal frequency (semi-solid food at least twice a day for breastfed infants 6–8 months and at least three times a day for breastfed children 9–23 months)).

In terms of mass media exposure, 11.1%, 3.3%, 48.5% and 8.8% mothers reported having high exposure to newspaper or magazine, radio, television, and movies, respectively. (Table 1) However, a notable 22.5% of mothers reported no exposure to any type of mass media. This lack of media exposure was even more pronounced in rural areas, where 29% of mothers had no media exposure, while in urban areas, only 8.8% of mothers lacked any media exposure. Evidently, in India, penetration of all four forms of mass media is higher in urban areas compared to rural areas, and television penetration and reach are broad compared to other forms of mass media^25^.

### Mass media and no prelacteal feeding

**Figure 1:**
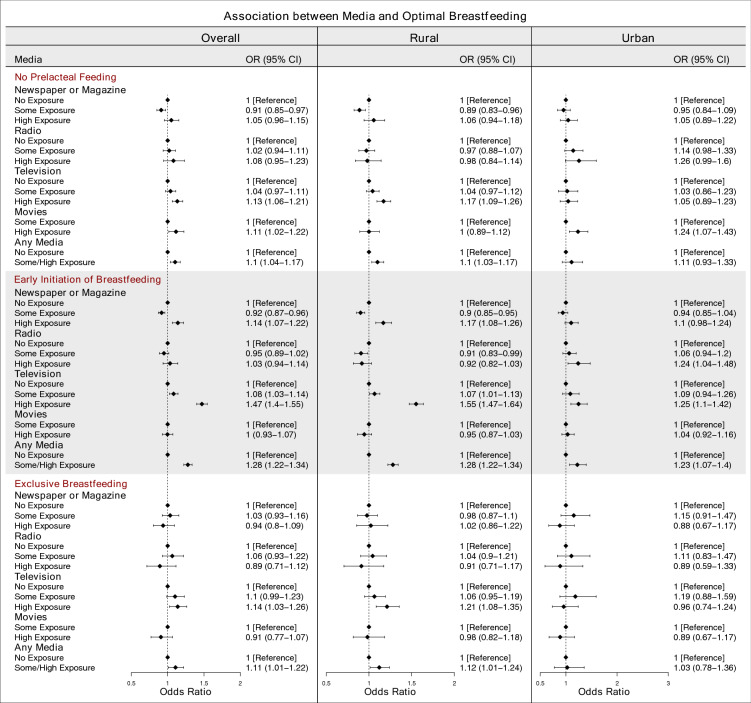
Association between different types of mass media and optimal breastfeeding practices. The ORs are mutually adjusted for other media exposure variables as well as the covariates: age, educational attainment, wealth, caste and religion. The ORs for any media are adjusted for the covariates. (Sample sizes: No prelacteal feeding = 116,908, Early Initiation of Breastfeeding = 122,458, Exclusive Breastfeeding = 22,839).

After adjusting for other media variables and covariates, our study found that, overall, women who had some newspaper or magazine exposure (Odds Ratio (OR) 0.91; 95% Confidence Interval (CI) 0.85–0.97) were associated with lower odds of not giving prelacteal feed to their infants compared to women who had no newspaper or magazine exposure. In contrast, women who had high television exposure (OR 1.13; 95% CI 1.06–1.21) and high movies exposure (OR 1.11; 95% CI 1.02–1.22) were associated with higher odds of not giving prelacteal feed to their infant compared to women who had no exposure to these TV and movies. After adjusting for the covariates, women who had some/high exposure to any media (OR 1.1; 95% CI 1.04–1.17) were associated with higher odds of not giving prelacteal feed to their infant compared to women who had no exposure to any media (Fig. 1).

In the rural areas, women who had some newspaper or magazine exposure (OR 0.89; 95% CI 0.83–0.96) were associated with lower odds of not giving prelacteal feed to their infants, and women who had high television exposure (OR 1.17; 95% CI 1.09–1.26) or some/high exposure to any media (OR 1.1; 95% CI 1.03–1.17) were associated with higher odds of not giving prelacteal feed to their infants compared to women who had no exposure to these media types. In the urban areas, women who had high movies exposure (OR 1.24; 95% CI 1.07–1.43) were associated with higher odds of not giving prelacteal feed to their infants compared to women who had no exposure to these media types.

### Mass media and early initiation of breastfeeding

After adjusting for other media variables and covariates, overall, women who had some newspaper or magazine exposure (OR 0.92; 95% CI 0.87–0.96) were associated with lower odds of EIBF compared to women who had no newspaper or magazine exposure. Women who had high newspaper or magazine exposure (OR 1.14; 95% CI 1.07–1.22), some television exposure (OR 1.08; 95% CI 1.03–1.14) and high television exposure (OR 1.47; 95% CI 1.4–1.55) were associated with higher odds of EIBF compared to women who had no exposure to these media types. After adjusting for the covariates, women who had some/high any media exposure (OR 1.28; 95% CI 1.22–1.34) were associated with higher odds of EIBF compared to women who had no exposure to any media (Fig. 1).

In the rural areas, women who had some newspaper or magazine exposure (OR 0.9; 95% CI 0.85–0.95) and some radio exposure (OR 0.91; 95% CI 0.83–0.99) were associated with lower odds of EIBF compared to women who had no exposure to these media types. Women who had some (OR 1.07; 95% CI 1.01–1.13) and high television exposure (OR 1.55; 95% CI 1.47–1.64) were associated with higher odds of EIBF compared to no television exposure. Moreover, women who had some/high any media exposure (OR 1.28; 95% CI 1.22–1.34) were associated with higher odds of EIBF compared to women who had no exposure to any media. In the urban areas, women who had high radio exposure (OR 1.24; 95% CI 1.04–1.48), high television exposure (OR 1.25; 95% CI 1.1–1.42) and some/high any media exposure (OR 1.23; 95% CI 1.07–1.4) were associated with higher odds of EIBF compared to women who had no exposure to these media types.

### Mass media and exclusive breastfeeding

After adjusting for other media variables and covariates, overall, women who had high television exposure (OR 1.14; 95% CI 1.03–1.26) were associated with higher odds of EBF compared to women who had no television exposure. After adjusting for the covariates, women who had some/high any media exposure (OR 1.11; 95% CI 1.01–1.22) were associated with higher odds of EBF compared to women who had no exposure to any media. (Fig. 1).

In the rural areas of India, women who had high television exposure (OR 1.21; 95% CI 1.08–1.35) and some/high any media exposure (OR 1.12; 95% CI 1.01–1.24) were associated with higher odds of EBF compared to women who had no exposure to these media types.

### Mass media and timely introduction of complementary foods

**Figure 2:**
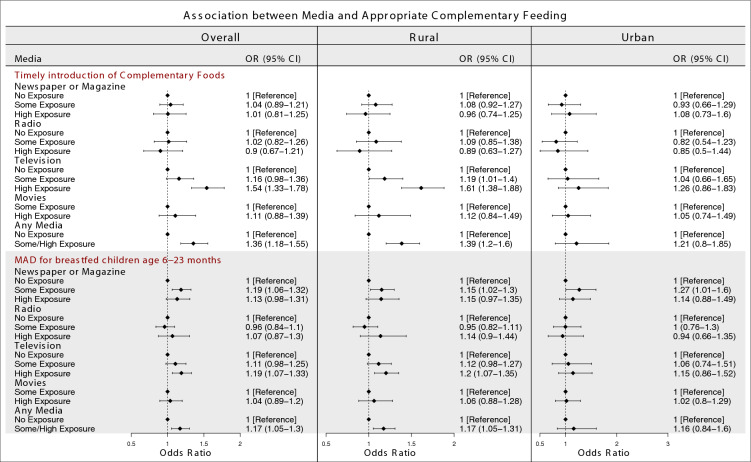
Association between different types of mass media and appropriate complementary feeding practices. The ORs are mutually adjusted for other media exposure variables as well as age, educational attainment, wealth, caste and religion. The ORs for any media are adjusted for the covariates. (Sample sizes: Complementary feeding = 10,809, Breastfeeding with MAD = 53,144).

After adjusting for other media variables and covariates, overall, women who had high television exposure (OR 1.54; 95% CI 1.33–1.78) were associated with higher odds of timely introducing complementary foods compared to women who had no television exposure. After adjusting for the covariates, women who had some/high any media exposure (OR 1.36; 95% CI 1.18–1.55) were associated with higher odds of timely introducing complementary foods compared to women who had no exposure to any media. (Fig. 2).

In the rural areas, women who had some television exposure (OR 1.19; 95% CI 1.01–1.4) and high television exposure (OR 1.61; 95% CI 1.38–1.88) were associated with higher odds of timely introducing complementary foods compared to women who had no television exposure. Women who had some/high any media exposure (OR 1.39; 95% CI 1.2–1.6) were associated with higher odds of timely introducing complementary foods compared to women who had no exposure to any media.

### Mass media and MAD for breastfed children ages 6–23 months

After adjusting for other media variables and covariates, our study also found that, overall, women who had some newspaper or magazine exposure (OR 1.19; 95% CI 1.06–1.32) and high television exposure (OR 1.19; 95% CI 1.07–1.33) were associated with higher odds of MAD for breastfed children ages 6–23 months compared to women who had no exposure to these media types. After adjusting for the covariates, women who had some/high any media exposure (OR 1.17; 95% CI 1.05–1.3) were associated with higher odds of MAD for breastfed children ages 6–23 months compared to women who had no exposure to any media.

In the rural areas, women who had some newspaper or magazine exposure (OR 1.15; 95% CI 1.02–1.3), high television exposure (OR 1.2; 95% CI 1.07–1.35) and some/high any media exposure (OR 1.17; 95% CI 1.05–1.31) were associated with higher odds of MAD for breastfed children ages 6–23 months compared to women who had no exposure to these media types. In the urban areas, women who had some newspaper or magazine exposure (OR 1.27; 95% CI 1.01–1.6) were associated with higher odds of MAD for breastfed children ages 6–23 months compared to women who had no newspaper or magazine exposure.

## Discussion

Mass media are effective means of communicating health messages because of their potential to reach large audiences across boundaries, especially through underserved rural areas^23,32^. Routine exposure to mass media can shape public knowledge, attitudes, beliefs, and behaviors and encourage healthy behavioral changes^22^. The use of mass media campaigns for health promotion and disease prevention have resulted in mixed evidence for success^23^. However, campaigns aimed towards increasing the adoption of IYCF practices have been successful^33,34^. The descriptive characteristics of our study established the popularity of different types of mass media amongst our target population, which indicates that television has the maximum penetration followed by newspaper and magazines. Television and cinema have their own advantages when it comes to creating health awareness. Audio-visual media can deliver messages to the target audience without a literacy or geographic barriers. However, accessibility of television allows for a much wider reach compared to cinema. Understandably, mass media, especially television, have been a crucial channel for health intervention programs to disseminate nutrition information in India^29,35^.

In recent decades, many efforts have gone towards improving the rate of IYCF practices in India. The Food Nutrition Board, Ministry of Women and Child Development (MoWCD), Government of India (GoI), developed the National Guidelines on IYCF in 2004 and updated them in 2006, to reach the national goals for the optimal IYCF practices^35^. These guidelines were developed in congruence with the WHO/UNICEF Global Strategy on IYCF^36^. Under these guidelines, mass media were used to create a climate of nutritional awareness in the country by launching special programs on IYCF^37^. Between 2004 and 2006, advertising campaigns on nutritional issues, like IYCF, were released in different regional languages, including 27 video films telecasted by *Doordarshan* (an Indian public service broadcaster and one of the largest broadcasting organizations), a 30-episode radio program and, posters and charts^30^. In 2013, the Ministry of Health and Family Welfare (MoHFW), GoI also created an operational guideline to guide the interventions on optimal IYCF practices, and further enhance these practices in India^38^. In 2016, the MoWCD launched a program to support breastfeeding called the Mother’s Absolute affection or MAA, where the focus was on the importance of the first 1000 days, thereby recognizing the importance of improving IYCF practices^35^. The program’s objectives include generation of breastfeeding awareness to facilitate higher breastfeeding rates in the country. To achieve maximum penetration of breastfeeding messages, the program used mass media, such as print, electronic and audio-visual platforms, amongst other communication channels like interpersonal communication. Most recently, POSHAN *Abhiyaan* or the National Nutrition Mission was launched in 2018 by MoWCD, GoI. This mission aims to improve nutritional outcomes and build knowledge, attitudes, and behavioral intent about overall nutrition, including optimal IYCF practices, amongst their target populations through a *Jan Andolan* or mass movement by utilizing platforms like mass media. In our study, we observed a positive association between exposure to at least one of the four mass media sources and all five IYCF practices in rural areas and EIBF in urban areas. This suggests that mass media exposure has the potential to encourage mothers to adopt healthy IYCF practices, especially in rural areas.

Over 66% of women in India have no exposure to newspapers or magazines, 22% women have had no schooling and 28% of women are not literate^39^. These numbers are even higher in the rural areas. In our study, nationally and in rural areas, high newspaper or magazines exposure was associated with EIBF. Some newspaper or magazines exposure was associated with providing MAD to breastfeeding children between 6 and 23 months in both rural and urban areas. However, newspaper or magazine exposure was not associated or was negatively associated with other IYCF practices. This suggests that while disseminating information via print media has the potential to influence mothers to practice appropriate IYCF, it has limitations in terms of reach since it excludes audiences with low literacy levels. Using visual aids to disseminate information via print media may allow women to overcome the literacy barrier and access more information through print media^40^.

**Figure 3:**
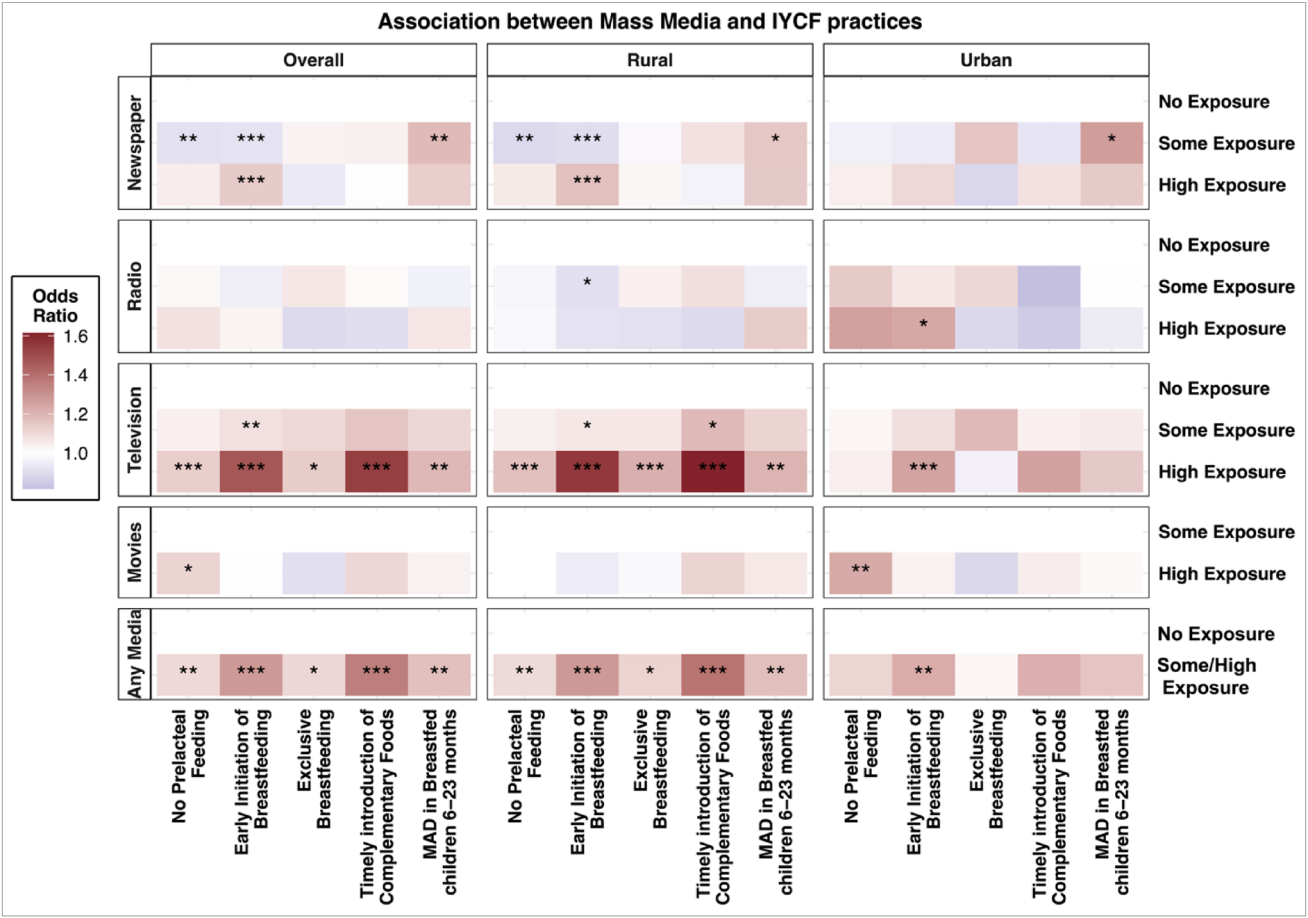
Overview of the associations between different types of mass media and appropriate IYCF practices. The associations are mutually adjusted for other media exposure variables as well as age, educational attainment, wealth, caste and religion. The associations for any media are adjusted for age, educational attainment, wealth, caste and religion. [*p < 0.05. **p < 0.01. ***p < 0.001].

Previous studies have found radio exposure to be associated with higher rates of EIBF^21^. In our study, high radio exposure was positively associated with EIBF in the urban areas. As per NFHS-5 data, more women in urban areas listen to the radio than in the rural areas^39^. Our findings suggest that radio could be a good medium to create awareness about IYCF practices, especially when targeted to urban areas (Fig. 3).

In a developing country like India, where percentage of illiteracy is high, television has a major role to play. Television is an equalizer in the context of health communication because it does not differentiate between audiences based on literacy. Moreover, the ubiquity of television exposure amongst our target population makes it an excellent medium to communicate health information. In our study, we found that even though more women in urban areas have exposure to television, television exposure was positively associated with all five IYCF practices in rural areas. Women in the rural areas of India have lower literacy levels compared to urban areas, likely due to limited access to schools and resources^41^. This suggests that disseminating messages to increase awareness about the benefits of IYCF practices via television could positively impact IYCF behaviors, especially among audiences with limited privileges. According to prior studies, when television is used as a medium to create knowledge, knowledge gaps resulting from the communication flow of messages are less likely to occur^42^. Our study suggests that using television as a medium to create awareness about IYCF practices, especially in the rural areas, could successfully promote the recommended IYCF behaviors. These findings complement our previous study that documents the effect of television on promoting maternal healthcare utilization in India^25^.

Our study also found significant associations between high movies exposure and no prelacteal feeding- nationally and in urban areas. As per NFHS-5 data, more women in urban areas than in the rural areas went to see movies in a cinema hall. This could be attributed to easier access to cinema halls in the urban areas^43^. Our findings suggest that messages disseminated via the cinema halls through advertisements, public service announcements or movies could create awareness about these practices, especially when targeted to the urban populations (Fig. 3).

Even though the penetration of mass media is higher in the urban areas, mass media exposure was associated with only a few optimal IYCF practices with no clear trend. Studies have found that the impact of mass media is greater among mothers in rural areas who have lower levels of education and other privileges^44^. Mothers with formal education have more knowledge about health, nutrition, and have a care-seeking behavior, which promotes the use of modern health services^45^. Moreover, the urban population could have better access to health-related information from other media sources, such as social media, as a higher proportion of women in urban areas than in rural areas have access to mobile phones and use the internet^39^. Hence, for them, mass media may not play a significant role in increasing awareness about maternal and child health practices.

According to the integrated theory of behavioral prediction, there are many factors that can impact the success of a mass media campaigns and lead to the desired health outcomes. For instance, an understanding of the determinants that can influence a health behavior is critical for the design and strategy of the campaign^24^. Campaigns will be most effective in influencing the human behavior by customizing health messages to influence the determinants leading to that behavior^28^. For instance, mass media campaigns have been found to increase the rate of EBF by promoting benefits of EBF in the first six months, highlighting the risks of giving water and other breastmilk substitutes, and also promoting positive social norms related to the practice^34^. Some authors also suggest that mass media alone may not be effective in influencing a behavior change and that combining mass media interventions with interpersonal counselling might be more conducive to positively influence IYCF behaviors^34,46^. Therefore, there could be additional barriers that need to be addressed to initiate a large change in social norms or cultural habits and improve the rate of recommended IYCF practices in India. Designing effective public health campaigns and combining mass media campaigns with additional interventions may improve the likelihood of success in future efforts to promote IYCF practices. Media campaigns, in complement with other strategic interventions at multiple levels may also reduce communication inequalities and promote healthy behavior changes^47,48^.

Our study is not without limitations. First, the cross-sectional design of the data prevents us from making causal inferences on the relationship between our exposure and outcome variables. However, the strong associations between media exposure and IYCF practices suggests the potential promise of media in promotion of appropriate IYCF practices. Second, NFHS data are self-reported. Thus, mothers who might be aware of the appropriate IYCF practices but did not practice them could have falsely reported their IYCF behaviors, leading to social desirability bias^49^. The self-reported nature of the data also makes our study susceptible to recall bias. Therefore, to minimize recall bias, we limited the age of the children while calculating the IYCF indicators. Finally, the NFHS-5 survey did not document frequency of mass media exposure through internet, social media, and mobile phones. Future surveys should also include information regarding internet, social media and mobile use and their implications on health-related behaviors. And future studies should examine the associations between these types of media and IYCF practices in India. NFHS-5 provides no information about the type of IYCF-related media content women in India are exposed to, thus, one can only speculate based on literature. Future studies should examine IYCF content (or lack thereof) in the media to obtain a complete picture of the kind of information encouraging (or not influencing) such behaviors.

## Conclusion

This study strengthens our understanding of the associations between different types of mass media and the optimal IYCF practices in India and provides evidence that mass media may promote healthy IYCF behaviors. Appropriately selecting the type of mass media to create awareness about different IYCF practices, in specific urban–rural settings, could help customize intervention programs to successfully influence IYCF behaviors. However, additional interventions like overcoming literacy barriers, interpersonal counselling and policy support may also be required to initiate large changes in social norms and cultural habits associated with some of these practices.

## Data Availability

The dataset supporting the conclusions of this article is available in the Demographic and Health Surveys Program repository (https://dhsprogram.com).
